# Supplementary material for: Validation of the Farsi Version of the Adult Concentration Inventory for Assessing Cognitive Disengagement Syndrome
Source: J Clin Med. 2023 Jul 11;12(14):4607. doi: 10.3390/jcm12144607 (PMC10380426; doi:10.3390/jcm12144607)
Supplement: Supplementary file 1 [file jcm-12-04607-s001.zip › jcm-2450106-supplementary.pdf]

تاریخ : ..../........./.......   نام و نام خانوادگی...

پرسشنامه تمرکز بزرگسالان (ACI)

لطفا جملات زیر را با دقت بخوانید و بگویید طی شش ماه گذشته هر کدام تا چه اندازه در مورد شما صادق بوده است.

شماره	سوالات	هیچوقت	گاهی وقتها	بیشتر وقتها	همیشه
۱	در انجام دادن کارها کند هستم.	۰	۱	۲	۳
۲	سردرگم هستم.	۰	۱	۲	۳
۳	به یک نقطه خیره می مانم.	۰	۱	۲	۳
۴	در طول روز بی حوصله هستم.	۰	۱	۲	۳
۵	خیال پردازی می کنم.	۰	۱	۲	۳
۶	به آسانی حواسم پرت می شود.	۰	۱	۲	۳
۷	فرد فعالی نیستم(میزان فعالیتم کم است).	۰	۱	۲	۳
۸	هنگام فکر کردن سردرگم هستم.	۰	۱	۲	۳
۹	به آسانی خسته می شوم.	۰	۱	۲	۳
۱۰	هنگام حرف زدن، حرفم را فراموش می کنم.	۰	۱	۲	۳
۱۱	احساس می کنم گیج شده ام.	۰	۱	۲	۳
۱۲	انگیزه کافی برای انجام کارهایم را ندارم.	۰	۱	۲	۳
۱۳	متوجه دنیای اطرافم نیستم.	۰	۱	۲	۳
۱۴	به آسانی قاطی می کنم.	۰	۱	۲	۳
۱۵	در فکر کردن کند هستم.	۰	۱	۲	۳
۱۶	بیان کردن فکرهایم برایم مشکل است(برایم سخت است فکرهایم را به زبان بیاورم).	۰	۱	۲	۳

رفتار ها ی ذکر شده در بالا تا چه اندازه منجر به ایجاد مشکل در موارد زیر می شود ؟

		اصلا	بسیار کم	کم	متوسط	زیاد	مصداق ندارد
۱۷	در شغل و محل کار	۰	۱	۲	۳	۴	
۱۸	در تحصیل و فعالیت های آموزشی	۰	۱	۲	۳	۴	
۱۹	در برقراری ارتباط با دوستان	۰	۱	۲	۳	۴	
۲۰	در برقراری ارتباط عاطفی	۰	۱	۲	۳	۴	
۲۱	در فرزند پروری	۰	۱	۲	۳	۴	
۲۲	تنظیم فعالیت های روز مره تان	۰	۱	۲	۳	۴	
۲۳	بهداشت فردی (حمام کردن، آراستگی، ورزش، تغذیه)	۰	۱	۲	۳	۴	
۲۴	در بهره مندی از خواب مناسب در طول شب	۰	۱	۲	۳	۴	

© Becker, S. P., Burns, G. L., & Willcutt, E. G.
© Farsi Version: Dena Sadeghi Bahmani.
